# Echocardiographic video-driven multi-task learning model for coronary artery disease diagnosis and severity grading

**DOI:** 10.3389/fbioe.2025.1556748

**Published:** 2025-07-25

**Authors:** Ying Guo, Yu-Han Cai, Tao Xu, Xin-Yang Song, Hong-Xia Guo, Min Dong, Dong Ni, Hui Li, Fang Wang, Wu-Feng Xue

**Affiliations:** ^1^ Department of Cardiology, Beijing Hospital, National Center for Gerontology, Institute of Geriatric Medicine, Chinese Academy of Medical Sciences, Beijing, China; ^2^ Medical Ultrasound Image Computing (MUSIC) Laboratory, School of Biomedical Engineering, Shenzhen University Medical School, Shenzhen University, Shenzhen, China; ^3^ Department of Echocardiography, State Key Laboratory of Cardiovascular Disease, Fuwai Hospital, National Center for Cardiovascular Diseases, Chinese Academy of Medical Sciences and Peking Union Medical College, Beijing, China

**Keywords:** coronary artery disease, stenosis, echocardiography, deep learning, strain, myocardial work

## Abstract

**Introduction:**

Echocardiography is a first-line noninvasive test for diagnosing coronary artery disease (CAD), but it depends on time-consuming visual assessments by experts.

**Methods:**

This study constructed an echocardiographic video-driven multi-task learning model, denoted Intelligent echo for CAD (IE-CAD), to facilitate CAD screening and stenosis grading. A 3DdeeplabV3+ backbone and multi-task learning were simultaneously incorporated into the core frame of the IE-CAD model to capture the dynamic myocardial contours. Multifarious features reflecting local semantic structures were extracted and integrated to yield echocardiographic metrics such as ejection fraction, strain, and myocardial work. For model training and testing, we used a total of 870 echocardiographic videos from 290 patients with clinically suspected CAD at Beijing Hospital (Beijing, China), split at an 8:2 ratio. To evaluate the model's generalizability, we used an external dataset comprising 450 echocardiographic videos from 150 patients at Fuwai Hospital (Beijing, China).

**Results:**

The IE-CAD model achieved an AUC of 0.78 and a sensitivity of 0.85 for detecting significant or severe CAD, with a pearson correlation coefficient of 0.545 for predicting the Gensini score. When applied to the external dataset, the model achieved an AUC of 0.77 and a sensitivity of 0.78 for detecting significant or severe CAD.

**Discussion:**

Thus, the IE-CAD model demonstrated effective CAD diagnosis and grading in patients with clinical suspicion.

**Trial registration:**

This work was registered at ClinicalTrials.gov on 05 April 2019 (registration number: NCT03905200).

## 1 Introduction

Coronary artery disease (CAD), a common ischemic heart disease, is a leading cause of death worldwide. CAD is caused by the buildup of fats and cholesterol in the walls of the coronary arteries. This process leads to a narrowing or blockage of the coronary arteries, resulting in heart ischemia. Early detection of CAD is vital to avoid myocardial infarction and improve prognostic outcomes of patients. CAD is diagnosed through a combination of physical exams, patient medical history, and a variety of tests such as electrocardiography, echocardiography, and coronary angiography. Coronary angiography, an invasive procedure that involves injecting dye into the coronary arteries and taking X-ray images, is considered the gold standard and definitive diagnostic test for CAD. Echocardiography is an ultrasound of the heart that provides information about coronary artery wall motion and chamber sizes. It is a non-invasive, cost-effective, and easily accessible imaging technique that is commonly used for CAD screening and evaluation (Task Force et al., 2013). However, the diagnosis by echocardiography is mostly grounded on visual assessment and requires considerable expertise. Emerging myocardial strain analysis by speckle tracking echocardiography (STE) has allowed a quantification of the active myocardial deformation and thereby expanded the echocardiographic approach for CAD detection to a new level (Malagoli et al., 2020). Nonetheless, STE still requires extensive experience in imaging data acquisition and interpretation.

With the advancement of Artificial Intelligence (AI) technology, machine learning algorithms have been used to build predictive models for CAD based on imaging and non-imaging Data (Muhammad et al., 2021; Zhang et al., 2021; Kigka et al., 2022). Currently, most AI-assisted diagnostic workflows for CAD are built upon computed tomography (CT), which has a number of disadvantages including radiation exposure, the use of contrast agent, and high cost (Chu et al., 2023; Ihdayhid et al., 2023). There have been few reports on CAD evaluation by AI-assisted echocardiography. In 2021, Salte and colleagues (Salte et al., 2021) constructed an AI model that was used to assess ventricular global longitudinal strain (GLS) based on STE videos. This model successfully performed automatic segmentation, motion estimates, and GLS measurements across various cardiac pathologies. In 2021, Upton et al. (2022) developed an AI-assisted image processing system to extract novel geometric and kinematic features from stress echocardiograms. This automated system identified patients with severe CAD with a specificity of 92.7% and a sensitivity of 84.4%, although disease severity was not quantified. Given that stress echocardiogram is a time-consuming test that may also have issues such as patient intolerance and poor image quality, it may not be suitable for early CAD screening and evaluation. Other advanced echocardiography techniques such as myocardial work have not yet been integrated with AI.

Traditional CAD diagnosis mainly relies on the percentage of coronary stenosis and often overlooks the anatomical complexity of the coronary tree. Relying solely on the degree of luminal narrowing may lead to underdiagnosis of the disease in certain patient populations (Mangla et al., 2017). The Gensini score takes into account the impact of the location of coronary stenosis on heart function and the cumulative effect of multivessel narrowing. As such, the Gensini score is commonly used to evaluate the severity of CAD (Rampidis et al., 2019). In this study, we developed an echocardiographic video-driven intelligent deep learning model for coronary stenosis evaluation and severity grading, which we named IE-CAD. In this model, transthoracic echocardiogram (TTE) videos of the standard apical 4-chamber (A4C), 3-chamber (A3C), and 2-chamber (A2C) views were used to calculate echocardiographic metrics including ejection fraction, GLS, and myocardial work, as well as the Gensini score that indicates the severity of coronary stenosis. We used this model to detect and evaluate CAD in subjects with clinically suspected CAD.

## 2 Materials and methods

### 2.1 Patients

**FIGURE 1:**
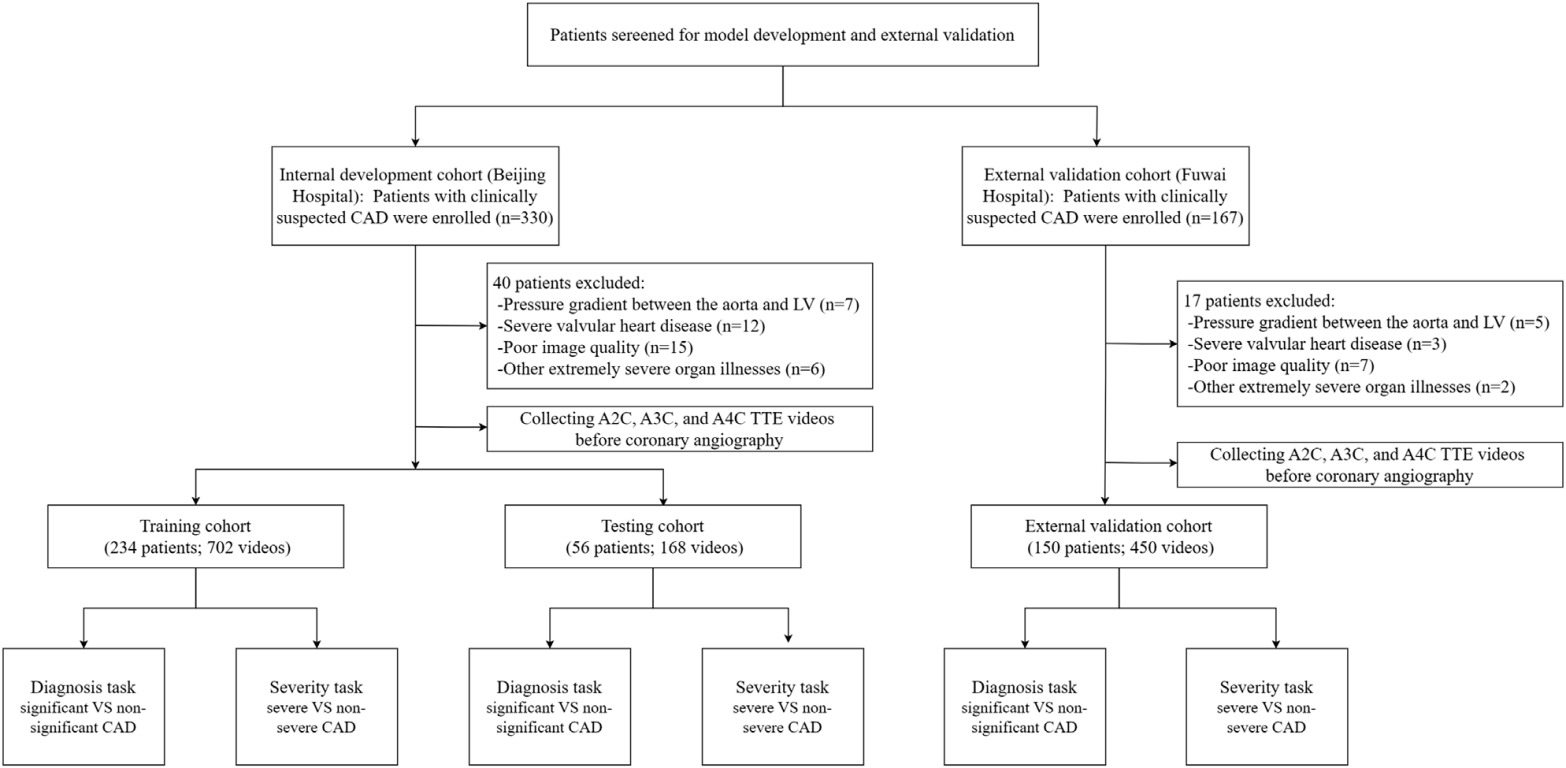
Workflow diagram of data collection.

This prospective, single-center clinical trial was registered at ClinicalTrials.gov on 05 April 2019. It represents a *post hoc* analysis of data from an IRB-approved prospective clinical trial with the registration number NCT03905200. Subject recruitment started in April 2019 and is still ongoing. Up to the time of the preparation of this article, the trial screened 330 subjects with clinically suspected CAD in Beijing hospital, Beijing, China. The inclusion criteria included (1) the presence of typical myocardial ischemia-related symptoms (e.g., shortness of breath, chest tightness, chest pain, and palpitation) or positive physical examination or blood test results, (2) the presence of sinus rhythm, and (3) 18 years of age or older. The exclusion criteria were (1) obstruction or pressure gradient between the aorta and left ventricle, (2) severe valvular heart disease or arrhythmia, (3) other extremely severe organ illnesses, and (4) poor image quality for speckle tracking. Out of the 330 subjects who were included in the initial screening, 40 were excluded and 290 were included in the data collection. The 290 subjects received baseline echocardiography 1 day before coronary angiography. A workflow chart of data collection is illustrated in Figure 1. We also included 150 subjects at Fuwai Hospital, Beijing, China according to the inclusion and exclusion criteria for external validation. This study was approved by our hospital Ethics Committee (reference number: 2020BJYYEC-021-02) and conducted in accordance with the Declaration of Helsinki. All subjects provided written informed consent.

### 2.2 Echocardiogram

Echocardiogram was performed in accordance with the Recommendations from the American Society of Echocardiography (Lang et al., 2015; Nagueh et al., 2016). Echocardiographic videos were captured on GE’s Vivid E9 and Vivid E95 ultrasound machines. The data were analyzed offline using the EchoPac software (EchoPAC v204, GE Vingmed Ultrasound, Norway). The cardiac images that showed the clearest visualization of the myocardium were selected for further analysis. Echocardiographic metrics were derived from the analysis of relevant features extracted from the videos. These metrics included left ventricular ejection fraction (LVEF) and factors related to strain and myocardial work. The Biplane Simpson’s method was used to calculate LVEF. The subjects were categorized into significant or non-significant CAD based on their extent of coronary stenosis. Significant CAD had a stenosis percentage of ≥50% for left main coronary artery or ≥70% for non-left main vessels (Lawton et al., 2022; Upton et al., 2022). All other subjects were categorized as “non-significant” CAD. The subjects were also categorized into severe or non-severe CAD based on their Gensini scores. The Gensini Scores were calculated using a modified method that incorporates lesion severity, location-specific multiplying factors, and collateral adjustments for total occlusions or near-total stenoses (Rampidis et al., 2019). The subjects with severe CAD had a Gensini score of >= 15 points, and the non-severe subjects had a Gensini score of <15 points. In accordance with prior studies (Wang et al., 2019; Yokokawa et al., 2020; Darand et al., 2023), the cutoff value of 15 was selected to balance sample sizes across groups and to avoid underestimating the condition of patients categorized as non-significant, favoring a lower cutoff to include more cases. The categorization criteria were determined by an adjudication committee made up of 3 blinded, unbiased experts, including 1 board-certified cardiologist.

### 2.3 Data set

All echocardiographic videos and images were stored in the core echocardiography laboratory of our hospital. The 290 individuals in the internal dataset were randomly divided into a training set of 234 subjects and a test set of 56 subjects (8:2). The test set matched the training set in terms of significant vs. non-significant and severe vs. non-severe CAD distribution (*P* > 0.1) so that both subsets accurately represented the overall distribution within the study cohort. The videos from the 150 subjects included in the external dataset were acquired on either a Philips EPIQ 7C or CVX ultrasound system—both different from the system used for the internal data—and were processed with institution-specific protocols. This external dataset was used to evaluate the model’s generalizability across clinical settings, equipment, and acquisition protocols.

### 2.4 Data pre-processing

The raw echocardiographic video data were cleaned, organized, and transformed into a format suitable for deep learning model training. The videos were first converted from a DICOM file into an AVI video file, containing the sector-shaped region only. Each frame and its corresponding annotated segmentation mask had 256 × 256 pixels with a native aspect ratio. Considering the variations in video frame rate and the subjects’ heart rate, we pre-processed video data of a complete cardiac cycle for each subject, including 10 frames from the end-diastole to end-systole and another 10 frames from the end-systole to end-diastole. This resulted in a 20 × 256 × 256 video block with corresponding segmentation masks for each subject.

### 2.5 Development of the IE-CAD model

**FIGURE 2:**
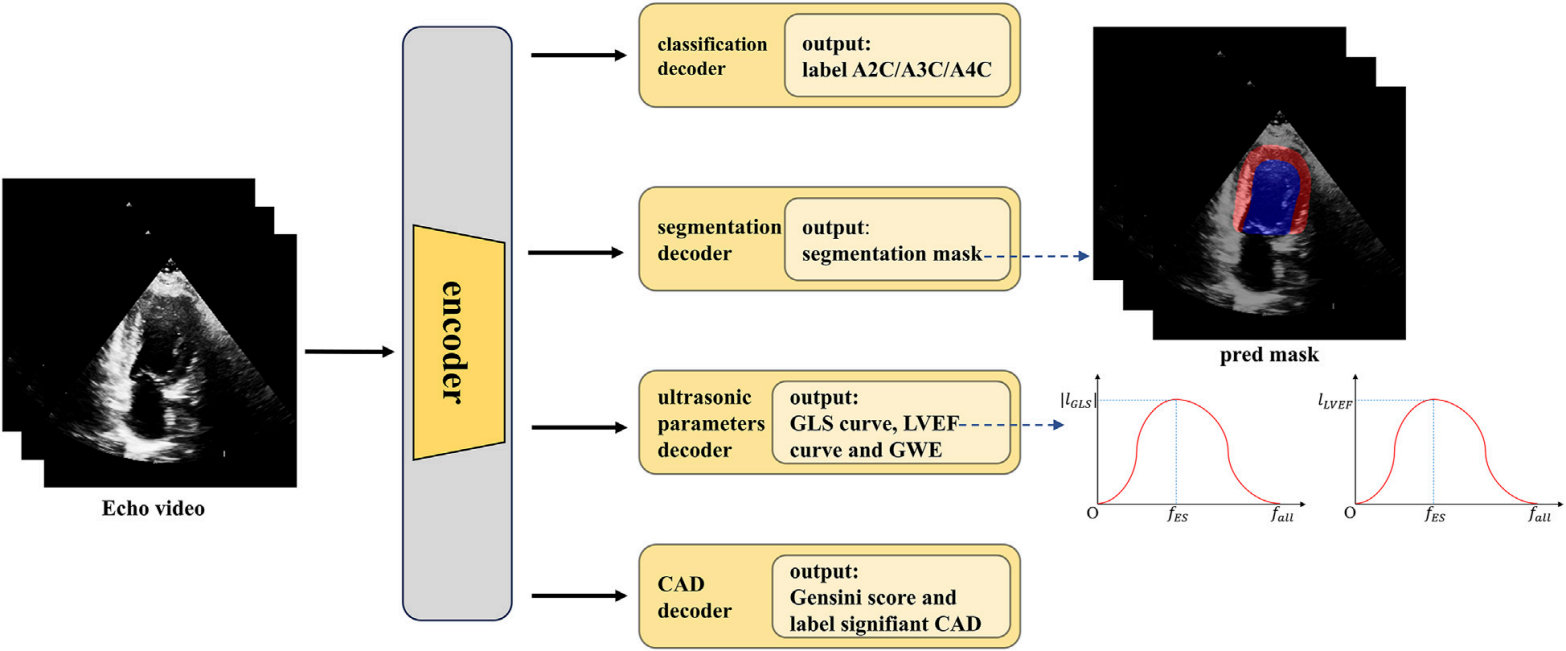
Methodology flowchart of the IE-CAD model.

A methodology flowchart of the IE-CAD model is illustrated in Figure 2. Firstly, low-level and deep semantic features were extracted from the echocardiographic videos through an encoder. The extracted image features were processed through a classification decoder, a segmentation decoder, an echocardiographic parameter decoder, and a CAD decoder to yield cross-sectional classification results, cardiac structure segmentation masks, GLS curves, LVEF curves, global work efficiency (GWE), Gensini scores, and significant or non-significant CAD classification results. Specifically, the low-level features were used for image classification, while the deep semantic features, in combination with the low-level features, were utilized for cardiac structure segmentation. The deep semantic features were also employed for the assessment of cardiac metrics including LVEF, GLS, and GWE. As a result, the extracted image features were enriched to represent local features of cardiac structures, as well as global features related to cardiac function. These local and global features were subsequently integrated for predictive tasks related to CAD detection and stenosis grading.

**FIGURE 3:**
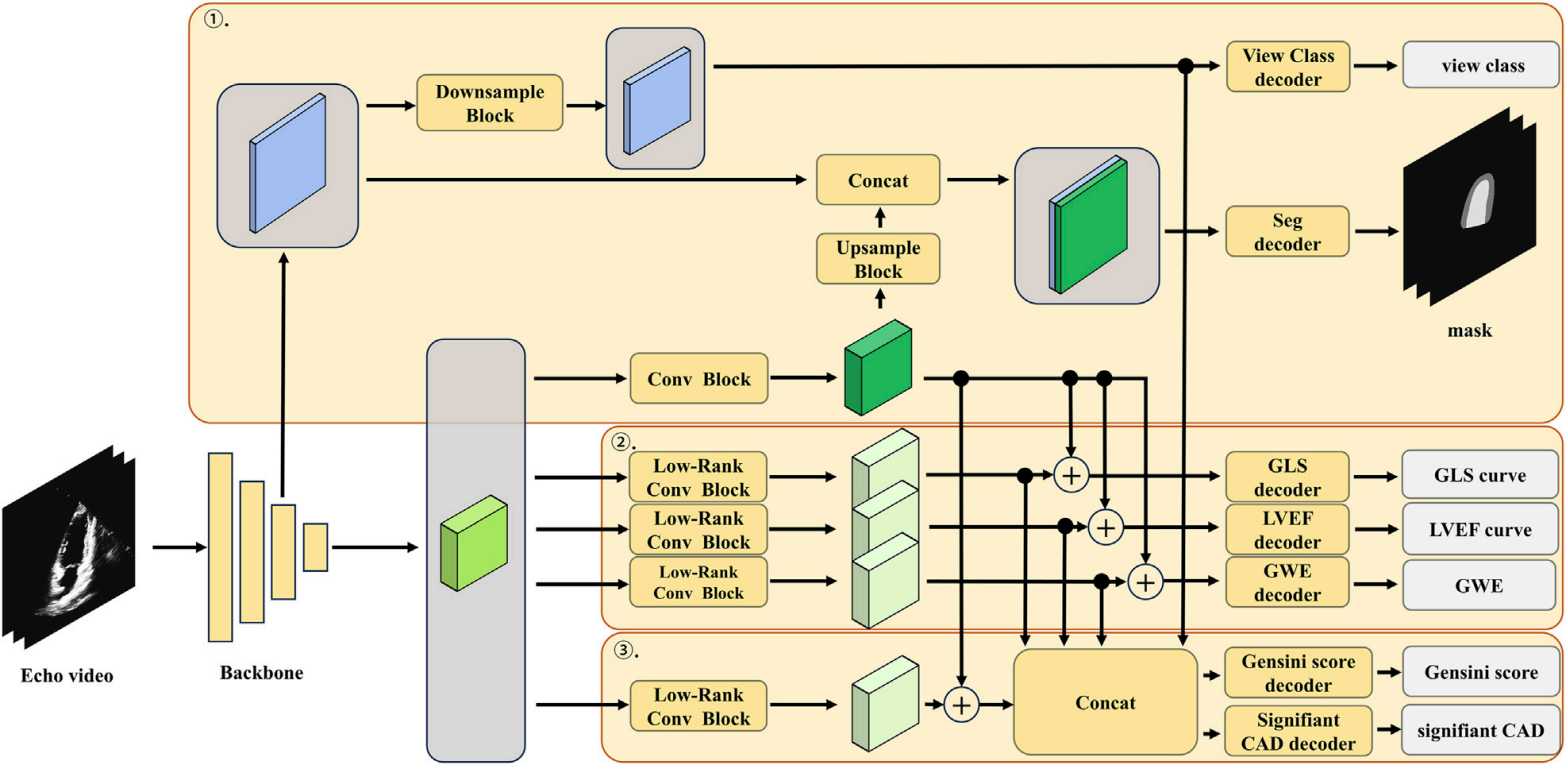
Architectural diagram of the multi-task learning model.

The detailed architecture of the low-rank multi-task deep learning model, including the number of layers, the number of filters in each convolutional layer, and the specific operations performed in each block, is illustrated in Figure 3; Table 1. The echocardiographic video clips were input into a 3D-DeepLabV3+ encoder backbone based on 3D-ResNet50 to generate low-level features (represented in blue in Figure 3) and deep semantic features (represented in green in Figure 3). The low-level features were first processed through a down-sampling network to match the dimensions of the deep semantic features and then through a view classification decoder to yield the view classification results. The deep semantic features were processed through a convolution block to generate the backbone features of the image (represented in dark green in Figure 3). After up-sampling, the backbone features were integrated with the low-level features before down-sampling, and the combined features were processed through a segmentation decoder to generate the segmentation masks of the ventricle and myocardium. The low-rank convolution block, compared to regular convolution block, can better extract sparse features, which can be utilized in multi-task learning to complement the primary features (Yang et al., 2024). The deep semantic features were further processed through three specific low-rank convolution blocks to extract sparse complementary features associated with GLS, LVEF, and GWE (represented in light green in Figure 3). The backbone and sparse features were integrated and processed through specific decoders to generate GLS, LVEF (in the form of curves), and GWE (in numerical values). The deep semantic features were again processed through a different low-rank convolution block to extract sparse complementary features associated with the Gensini score (represented in light green in Figure 3), which were fused with the backbone features, specialized features for GLS, LVEF, and GWE, and the low-level features used for view classification. The integrated features were processed through a score decoder and a CAD classification decoder to yield the Gensini score and CAD classification results, respectively.

**TABLE 1:** Specifications of all network module architectures of the IE-CAD model.

Module	Input size	Output size	Architecture
Conv Block	256 × 20 × 16 × 16	256 × 20 × 16 × 16	Conv3d (256, 256, 3 × 3 × 3), BN, ReLU Conv3d (256, 256, 1 × 1 × 1), BN, ReLU
Low-Rank Conv Block	256 × 20 × 16 × 16	256 × 20 × 16 × 16	Low-Rank Conv3d (256, 256, 3 × 3 × 3, r = 4), BN, ReLU Low-Rank Conv3d (256, 256, 1 × 1 × 1, r = 4), BN, ReLU
View class decoder	48 × 20 × 16 × 16	3 × 1	AvgPool3d (1, 1, 1), FC (48, 3), Softmax
Seg decoder	304 × 20 × 64 × 64	3 × 20 × 256 × 256	Conv3d (304, 256, 3 × 3 × 3), BN, ReLU Conv3d (256, 3, 1 × 1 × 1), Upsample, Softmax
GLS decoder	256 × 20 × 16 × 16	20 × 1	AvgPool3d (None, 1, 1) Bi-LSTM, FC (512, 1)
LVEF decoder	256 × 20 × 16 × 16	20 × 1	AvgPool3d (None, 1, 1) Bi-LSTM, FC (512, 1)
GWE decoder	256 × 20 × 16 × 16	1 × 1	AvgPool3d (1, 1, 1), FC (256, 1)
Gensini score decoder	1,072 × 20 × 16 × 16	1 × 1	Conv3d (1,072, 512, 3 × 3 × 3), BN, ReLU Temporal Attention (nhead = 8, dim = 512) Mean (1), FC (512, 256), GELU, FC (256, 1)
Significant CAD decoder	1,072 × 20 × 16 × 16	1 × 1	Conv3d (1,072, 512, 3 × 3 × 3), BN, ReLU Temporal Attention (nhead = 8, dim = 512) Mean (1), FC (512, 256), ReLU, FC (256, 2) Softmax

CAD, coronary artery disease; GLS, global longitudinal strain; GWE, global work efficiency; LVEF, left ventricular ejection fraction.

### 2.6 Model training, testing and external validation

This model was trained in three steps. The first step involved training on view classification and heart structure segmentation. The cross-entropy loss function was used for training on view classification, while both the cross-entropy loss and Dice loss functions were employed for training on heart structure segmentation. The second step involved training on regression models for estimating GLS, LVEF, and GWE using the mean squared error loss. The third step involved training on the network for CAD classification and Gensini score prediction while the backbone weight fractions of view classification, heart structure segmentation, and metrics regression remained constant. The cross-entropy loss and mean squared error loss were used for training on CAD classification and Gensini score prediction, respectively. The entire training process consisted of three sequential phases: 30 epochs in the first step, 30 epochs in the second step, and 90 epochs in the third step. The final training results were generated from the last epoch. The training was performed on two NVIDIA A6000 GPUs, using the Adam optimizer and following a cosine annealing schedule. Each time the training loss function was altered, the learning rate was re-warmed, starting with an initial rate of 2 × 10^−4^ and a weight decay of 1 × 10^−5^. The batch size was 16, with parameters updated after every three gradient accumulations.

During model testing, the A4C, A3C, and A2C views of each echocardiogram were tested separately. The final Gensini score was defined as the maximum predicted value from the three views. The case was classified as significant CAD if one or more views were classified as significant. This approach maximizes the model’s sensitivity to detect CAD and minimizes underdiagnosis due to variations between different views. The same methods were applied to the external dataset to validate the generalizability of the IE-CAD model.

### 2.7 Statistical analysis

Continuous variables with normal distribution are presented as the mean and standard deviation, whereas those not conforming to normal distribution are presented as the median and interquartile range. Categorical variables were compared using the Chi-square test or Fisher’s exact test. Comparative analysis of continuous variables was conducted utilizing the *t*-test or Mann-Whitney U test. The performance of diagnostic measures was indicated by 95% confidence intervals. This study used the Dice coefficient to quantify cardiac structure segmentation performance, a common metric in image segmentation that measures the overlap between binary masks. The Mean Absolute Error (MAE), a metric that calculates the average absolute errors between the predicted and actual values, was used to assess the performance of the regression models for EF, GLS, and GWE prediction, with lower scores reflecting lower prediction errors. The predictive performance for significant and/or severe CAD was evaluated in terms of accuracy, precision, sensitivity, specificity, and the F1 Score and through receiver operating characteristic (ROC) curve analysis. The intra- and inter-observer variability of the strain indices was gauged by intraclass correlation coefficients. Test outcomes with a *P*-value of less than 0.05 were considered significantly different. All statistical computations were executed utilizing SPSS software version 26.0.

## 3 Results

### 3.1 Clinical characteristics and echocardiographic metrics of CAD patients

The 290 subjects who were included in data collection had an average age of 62.9 ± 9.9 years. There were 45 (15.5%) women and 245 (84.5%) men. A total of 122 (42.1%) subjects were diagnosed with CAD by coronary angiography. Table 2 summarizes the demographic and clinical characteristics of the 122 CAD-positive and 168 CAD-negative subjects and their echocardiographic metrics estimated using the IE-CAD model. Compared with the CAD-negative subjects, the CAD-positive subjects had a substantially higher Gensini score (45.77 vs. 13.18, *P* < 0.001). They also had higher incidence rate of diabetes (51.6% vs. 38.7%, *P* = 0.028) and elevated plasma levels of HbA1c (6.66% vs. 6.31%, *P* = 0.004), brain natriuretic peptide (BNP) (92.14 pg/mL vs. 37.92 pg/mL, *P* = 0.004), and creatinine (88.63 μmol/L vs. 73.18 μmol/L, *P* = 0.037). There were no significant differences in other characteristics between the two groups, including age, gender, body mass index (BMI), family history of CAD, smoking, hyperlipidemia, systolic and diastolic blood pressures, suspected myocardial ischemia by electrocardiogram, fasting plasma glucose, uric acid and LDL cholesterol (*P* > 0.05).

**TABLE 2:** Summary of demographic and clinical characteristics of the CAD-positive and CAD-negative subjects and their echocardiographic metrics predicted by the IE-CAD model.

Characteristics	Total (n = 290)	CAD-negative (n = 168)	CAD-positive (n = 122)	*P*-value
Gensini score	26.89 ± 26.69	13.18 ± 13.56	45.77 ± 28.76	**<0.001** ^***^
Age, year	62.88 ± 9.92	62.71 ± 10.46	63.11 ± 9.16	0.731
Male, n (%)	245 (84.48)	137 (81.55)	108 (88.52)	0.105
Family history of CAD, n (%)	82 (28.28)	50 (29.76)	32 (26.23)	0.510
Diabetes mellitus, n (%)	128 (44.14)	65 (38.69)	63 (51.64)	**0.028** ^*^
Smoking, n (%)	181 (62.41)	99 (58.93)	82 (67.21)	0.150
Hyperlipidemia, n (%)	225 (77.59)	132 (78.57)	93 (76.23)	0.637
Hypertension, n (%)	207 (71.38)	120 (71.43)	87 (71.31)	0.983
BMI, kg/m^2^	25.37 ± 3.23	25.51 ± 3.38	25.17 ± 3.00	0.384
Systolic BP, mmHg	132.51 ± 17.02	133.27 ± 17.08	131.48 ± 16.95	0.377
Diastolic BP, mmHg	79.24 ± 48.67	81.55 ± 63.32	76.07 ± 10.35	0.344
Suspected myocardial ischemia by electrocardiogram, n (%)	90 (31.03)	46 (27.38)	44 (36.07)	0.115
BNP, pg/mL	60.73 ± 138.13	37.92 ± 49.27	92.14 ± 201.26	**0.004** ^**^
HbA1c, %	6.46 ± 1.03	6.31 ± 0.96	6.66 ± 1.09	**0.004** ^**^
Creatinine, μmol/L	79.68 ± 53.57	73.18 ± 14.03	88.63 ± 80.27	**0.037** ^*^
FPG, mmol/L	5.99 ± 1.75	5.83 ± 1.42	6.21 ± 2.11	0.064
Uric acid, μmol/L	348.02 ± 85.04	345.17 ± 85.53	351.95 ± 84.54	0.503
LDL-C, mmol/L	2.13 ± 0.86	2.10 ± 0.86	2.19 ± 0.87	0.385
Echocardiographic metrics				
Left atrial diameter, mm	35.06 ± 4.07	34.95 ± 4.15	35.20 ± 3.97	0.603
Interventricular septum end-diastolic thickness, mm	10.17 ± 1.18	10.11 ± 1.17	10.25 ± 1.19	0.296
Left ventricular end-diastolic diameter, mm	46.63 ± 4.76	46.29 ± 3.95	47.10 ± 5.66	0.175
Left ventricular posterior wall end-diastolic thickness, mm	9.90 ± 1.14	9.75 ± 1.11	10.11 ± 1.16	**0.007** ^**^
Left ventricular end-diastolic volume, mL	101.99 ± 23.15	99.80 ± 20.21	105.00 ± 26.47	0.070
Mitral E/e’ ratio	12.66 ± 4.57	12.25 ± 3.64	13.22 ± 5.57	0.095
LVEF, %	61.47 ± 6.77	63.17 ± 4.46	59.13 ± 8.52	**<0.001** ^***^
Regional wall motion abnormality, n (%)	35 (12.07)	10 (5.95)	25 (20.49)	**<0.001** ^***^
GLS, %	−18.43 ± 3.42	−20.11 ± 2.12	−16.11 ± 3.54	**<0.001** ^***^
PSD, ms	56.09 ± 23.54	50.10 ± 19.24	64.34 ± 26.35	**<0.001** ^***^
GWI, mmHg%	1872.49 ± 425.87	2023.61 ± 345.37	1,664.39 ± 439.53	**<0.001** ^***^
GCW, mmHg%	2,190.76 ± 486.64	2,374.68 ± 387.62	1937.50 ± 496.61	**<0.001** ^***^
GWW, mmHg%	124.39 ± 92.79	111.46 ± 86.65	142.20 ± 98.24	**0.005** ^**^
GWE, %	93.26 ± 4.95	94.65 ± 3.56	91.34 ± 5.89	**<0.001** ^***^
GPW, mmHg%	2099.77 ± 447.85	2,262.46 ± 368.27	1875.75 ± 452.23	**<0.001** ^***^
GNW, mmHg%	215.36 ± 90.63	223.68 ± 87.73	203.90 ± 93.64	0.066
GSCW, mmHg%	2063.90 ± 454.53	2,232.77 ± 365.47	1831.35 ± 463.65	**<0.001** ^***^
GSWW, mmHg%	88.55 ± 71.01	81.79 ± 66.55	97.87 ± 76.02	0.057

^*^ *P* < 0.05.

^**^ *P* < 0.01.

^***^ *P* < 0.001 compared with CAD-negative subjects.

All bolded results indicate *P* < 0.05, meaning that the differences between the CAD-negative and CAD-positive groups are statistically significant.

BMI, body mass index; BP, blood pressure; CAD, coronary artery disease; FPG, fasting plasma glucose; GCW, global constructive work; GLS, global longitudinal strain; GNW, global negative work; GPW, global positive work; GSCW, global systolic constructive work; GSWW, global systolic wasted work; GWE, global work efficiency; GWI, global work index; GWW, global wasted work; LDL-C, low-density lipoprotein cholesterol; LVEF, left ventricular ejection fraction; PSD, peak strain dispersion; TDI, tissue doppler imaging.

There were significant differences in most echocardiographic metrics between the two groups (*P* < 0.05), which included left ventricular posterior wall end-diastolic thickness, LVEF, regional wall motion abnormality, GLS, peak strain dispersion, global work index, global constructive work, global wasted work, GWE, global positive work, and global systolic constructive work. GLS is an indicator of the left ventricle deformation (strain) in the longitudinal direction (Voigt et al., 2015). Myocardial work incorporates both left ventricle deformation (strain) and afterload (pressure the heart must work against), allowing for a better evaluation of cardiac performance (Edwards et al., 2019; Guo et al., 2022). In regard to strain and myocardial work metrics, absolute values of GLS, global work index and GWE were significantly higher in non-significant CAD patients when compared to significant CAD patients (*P* < 0.001). These results supported the inclusion of GLS, global work index and GWE as assistant tasks in informative feature extraction for CAD evaluation. There were no significant differences between the two groups in left atrial diameter, left ventricular end-diastolic volume, and Mitral E/e’ ratio (*P* > 0.05), which were conventional echocardiographic indices.

### 3.2 Predictive performance of the IE-CAD model

#### 3.2.1 Gensini score prediction

**FIGURE 4:**
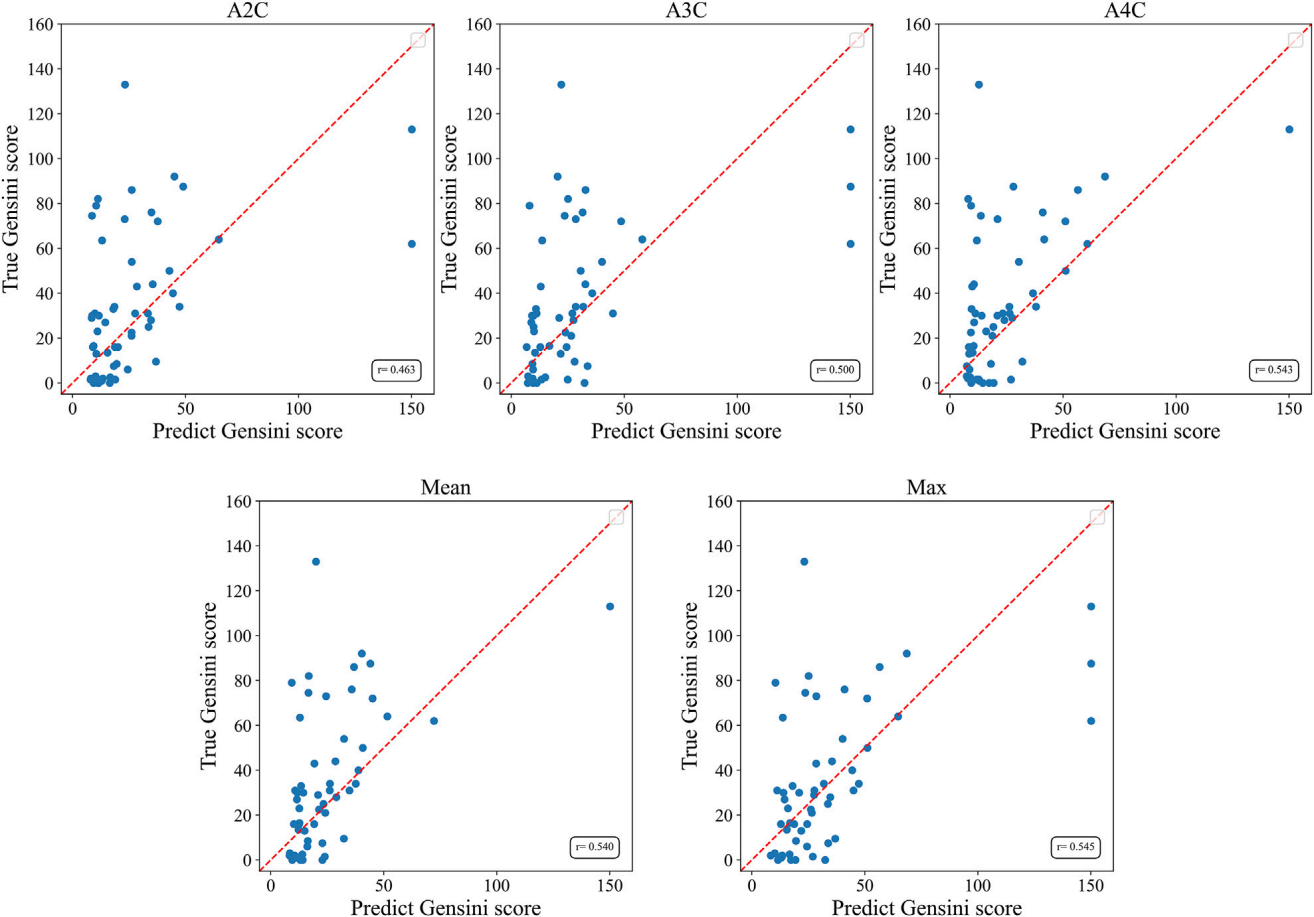
Scatter plots of Gensini scores predicted by the IE-CAD model vs. those determined by coronary angiography. Gensini label, Gensini scores by coronary angiography; Gensini pred, Gensini scores predicted by the IE-CAD model; A4C, the apical 4-chamber view; A3C, the apical 3-chamber view; A2C, the apical 2-chamber view; mean, mean Gensini scores predicted by the IE-CAD model across the A4C, A3C, and A2C views; max, maximal Gensini scores predicted by the IE-CAD model across the A4C, A3C, and A2C views.

The Gensini score prediction results of the IE-CAD model are shown in Figure 4; Table 3. The maximal predicted scores across the A4C, A3C, and A2C views showed a Pearson correlation coefficient of 0.545 (Table 3), while the mean predicted scores across the three apical chamber views showed a slightly lower Pearson correlation coefficient of 0.540. Thus, the maximal predicted scores were used for subsequent CAD classification.

**TABLE 3:** Performance of the IE-CAD model in Gensini score prediction.

View	MAE	PCC
A2C	22.0	0.463
A3C	22.2	0.500
A4C	19.8	0.542
Mean	19.7	0.540
Max	20.5	0.545

MAE, mean absolute error; PCC, pearson correlation coefficient; A2C, apical 2-chamber; A3C, apical 3-chamber; A4C, apical 4-chamber; Mean, mean value across the A2C, A3C, and A4C views; Max, maximal value across the A2C, A3C, and A4C views.

#### 3.2.2 Echocardiographic metrics prediction

**FIGURE 5:**
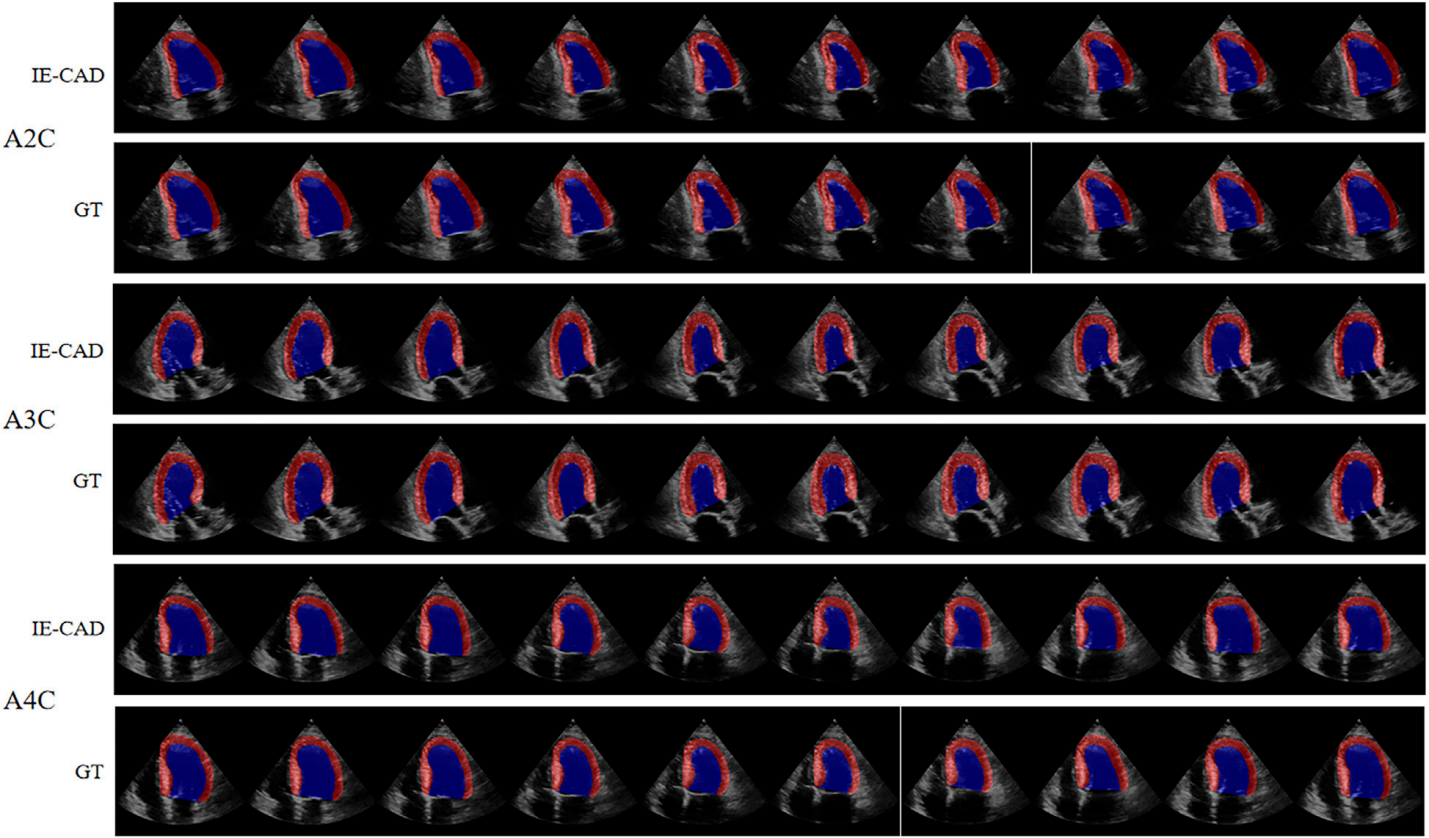
Sample Image of Cardiac Structure Segmentation. “A2C,” “A3C,” and “A4C” denote the corresponding standard image planes. The “IE-CAD” column displays the model’s predicted segmentation results, while the “GT” column shows the ground truth segmentation annotations. In the segmentation results, the red region indicates the predicted left myocardium, and the blue region indicates the predicted left ventricle.

The cardiac structure segmentation and echocardiographic metrics prediction performance results of the IE-CAD model are presented in Tables 4, 5. Figure 5 shows sample cardiac structure segmentation results. The model’s predictions exhibited strong agreement with the ground truth annotations and maintained good temporal continuity throughout the complete cardiac cycle. This demonstrates the model’s effective capture of the structural and motion characteristics of both the left myocardium and left ventricle, providing a solid foundation for subsequent CAD-assisted diagnostic predictions. The MAEs for predicting GLS, LVEF, and GWE were 1.88%, 5.11%, and 2.6%, respectively. Compared to AI-assisted regression black box models for echocardiography (Christensen et al., 2024; Kwan et al., 2024), the IE-CAD model was able to extract latent features related to GLS, LVEF, and GWE more effectively, thus achieving a better performance in capturing the temporal myocardium features. By integrating these features into cross-sectional classification, cardiac structure segmentation, and model-learned features pertinent to CAD diagnosis, the IE-CAD model was able to achieve high performance in Gensini score prediction and CAD classification.

**TABLE 4:** Performance of the IE-CAD model in cardiac structure segmentation.

	Dice value	
View	Left myocardium	Left ventricle
A2C	0.825	0.902
A3C	0.842	0.908
A4C	0.844	0.912
Mean	0.837	0.907

A2C, apical 2-chamber; A3C, apical 3-chamber; A4C, apical 4-chamber; Mean, mean value across the A2C, A3C, and A4C views.

**TABLE 5:** Performance of the IE-CAD model in echocardiographic metrics estimation.

Parameter	MAE
GLS (%)	1.88
LVEF (%)	5.11
GWE (%)	2.6

GLS, global longitudinal strain; LVEF, left ventricular ejection fraction; GWE, global work efficiency; MAE, mean absolute error.

#### 3.2.3 Detection of significant CAD and severe CAD

**FIGURE 6:**
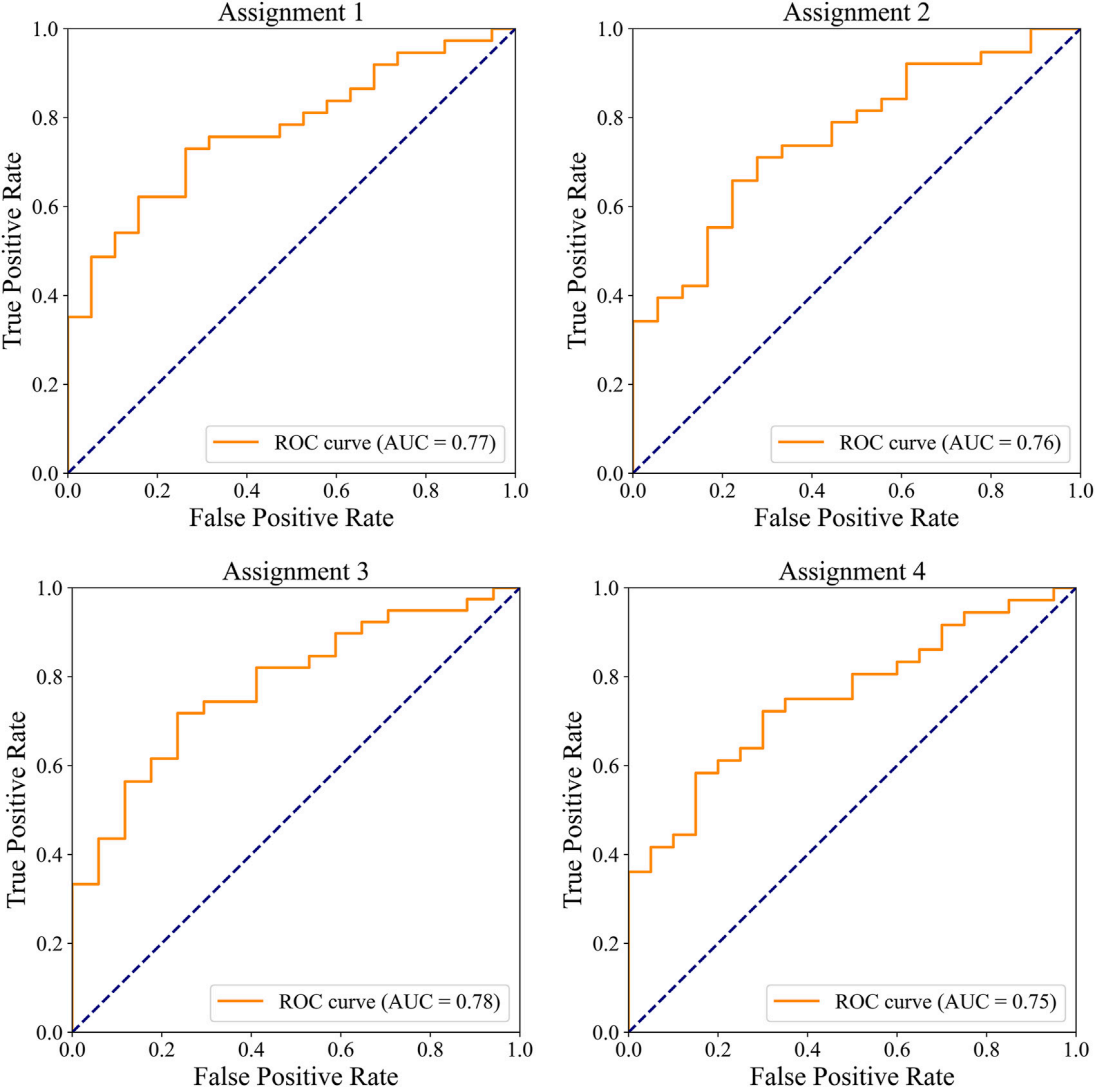
ROC curves illustrating the performance of the IE-CAD model in detecting significant CAD and/or severe CAD within the internal cohort.

The ROC curves for the detection of significant CAD and/or severe CAD (predicted Gensini score >= 15) by the IE-CAD model are presented in Figure 6. The detection performance results are presented in Table 6. The IE-CAD model detected significant CAD with an AUC of 0.77 and severe CAD with an AUC of 0.76. It detected cases that were classified as either significant or severe CAD but not both with an AUC of 0.78 and a sensitivity of 0.85. Finally, the model detected cases that were classified as both significant and severe CAD with an AUC of 0.75.

**TABLE 6:** Performance of the IE-CAD model for detecting significant CAD and/or severe CAD.

Assignment	Accuracy	Precision	Sensitivity	Specificity	F1 score	AUC
Assignment 1	0.73 (0.71, 0.75)	0.82 (0.80, 0.84)	0.76 (0.73, 0.79)	0.68 (0.64, 0.72)	0.79 (0.77, 0.81)	0.77 (0.76, 0.78)
Assignment 2	0.70 (0.66, 0.74)	0.74 (0.71, 0.77)	0.84 (0.83, 0.85)	0.39 (0.32, 0.46)	0.79 (0.77, 0.81)	0.76 (0.75, 0.77)
Assignment 3	0.71 (0.68, 0.74)	0.77 (0.75, 0.79)	0.85 (0.84, 0.86)	0.41 (0.34, 0.48)	0.80 (0.78, 0.82)	0.78 (0.77, 0.79)
Assignment 4	0.71 (0.68, 0.74)	0.79 (0.77, 0.81)	0.75 (0.72, 0.78)	0.65 (0.61, 0.69)	0.77 (0.75, 0.79)	0.75 (0.73, 0.77)

Assignment 1: Detection of cases classified as significant CAD.

Assignment 2: Detection of cases classified as severe CAD.

Assignment 3: Detection of cases classified as either significant CAD, or severe CAD but not both.

Assignment 4: Detection of cased classified as both significant CAD, and severe CAD.

#### 3.2.4 External validation of model performance

**FIGURE 7:**
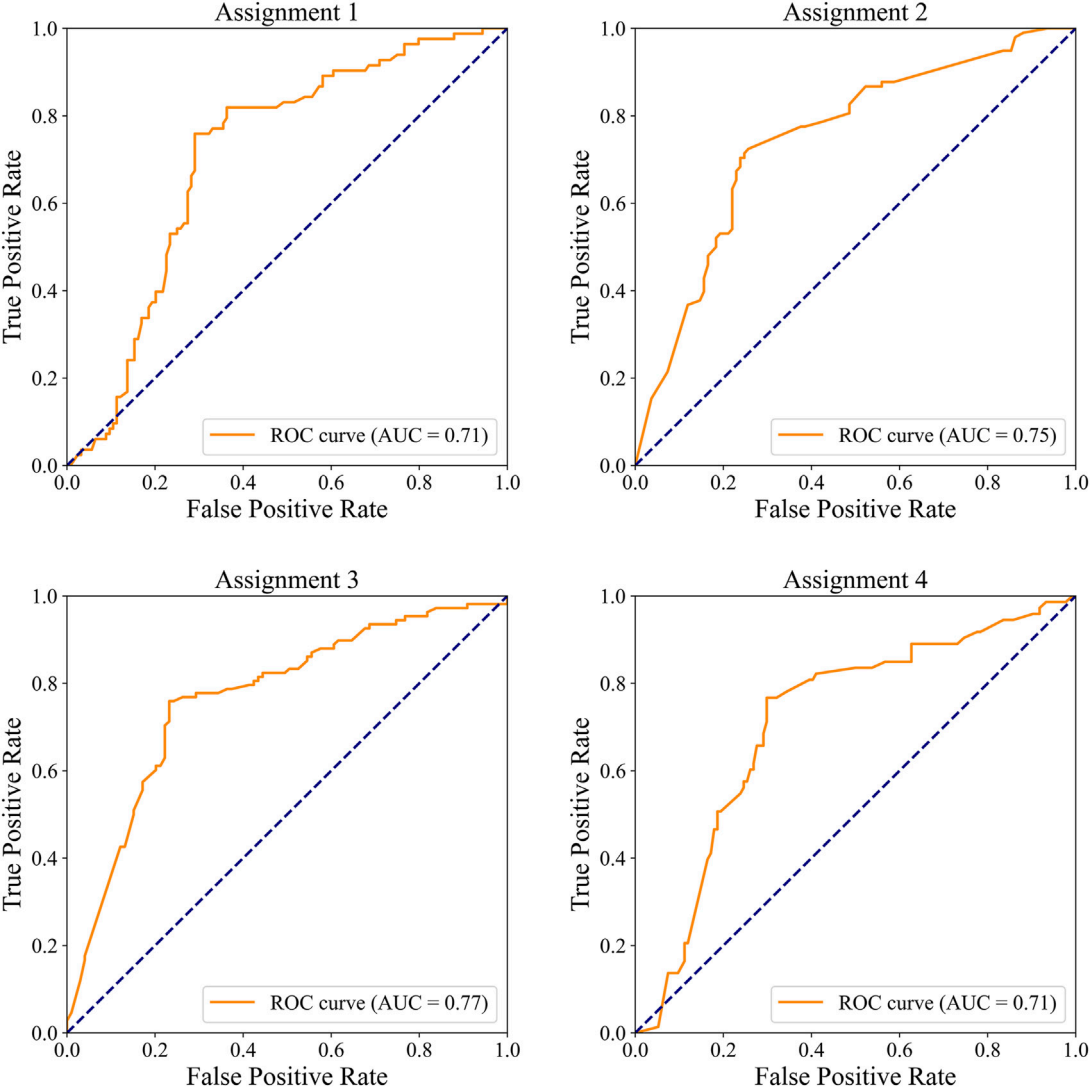
ROC curves illustrating the performance of the IE-CAD model in detecting significant CAD and/or severe CAD within the external validation cohort.

In the external dataset, the ROC curves for detecting significant CAD and/or severe CAD are presented in Figure 7, and the corresponding performance metrics are summarized in Table 7. The IE-CAD model achieved an AUC of 0.71 for detecting significant CAD and 0.75 for severe CAD. For cases classified as either significant or severe CAD (but not both), the model achieved an AUC of 0.77. For cases classified as both significant and severe CAD, the AUC was 0.71.

**TABLE 7:** Performance of the IE-CAD model in external validation.

Assignment	Accuracy	Precision	Sensitivity	Specificity	F1 score	AUC
Assignment 1	0.64 (0.61, 0.67)	0.54 (0.52, 0.56)	0.82 (0.81, 0.83)	0.52 (0.50, 0.54)	0.65 (0.63, 0.67)	0.71 (0.69, 0.73)
Assignment 2	0.73 (0.71, 0.75)	0.72 (0.70, 0.74)	0.71 (0.69, 0.73)	0.75 (0.73, 0.77)	0.72 (0.70, 0.74)	0.75 (0.73, 0.77)
Assignment 3	0.74 (0.72, 0.76)	0.74 (0.73, 0.75)	0.78 (0.76, 0.80)	0.71 (0.68, 0.74)	0.76 (0.74, 0.78)	0.77 (0.76, 0.78)
Assignment 4	0.72 (0.68, 0.76)	0.58 (0.55, 0.61)	0.77 (0.75, 0.79)	0.70 (0.67, 0.73)	0.66 (0.63, 0.69)	0.71 (0.68, 0.74)

Assignment 1: Detection of cases classified as significant CAD.

Assignment 2: Detection of cases classified as severe CAD.

Assignment 3: Detection of cases classified as either significant CAD, or severe CAD but not both.

Assignment 4: Detection of cased classified as both significant CAD, and severe CAD.

## 4 Discussion

In this study, an echocardiographic video-driven AI model denoted IE-CAD was established using a deep learning-based 3DdeeplabV3+ network for automatic myocardial motion estimation and cardiac structure segmentation. This model integrated traditional LVEF, GLS, and myocardial work measurements for CAD diagnosis and stenosis severity assessment. In a prospective single-center trial, the IE-CAD model automatically and accurately classified cardiac views, tracked myocardial motion, and estimated echocardiographic metrics such as GLS, LVEF, and GWE in subjects with clinically suspected CAD. This model detected significant CAD with an AUC of 0.77 and severe CAD with an AUC of 0.76, allowing rapid, noninvasive, and cost-effective CAD diagnosis and evaluation. As shown in Table 7; Figure 7, IE-CAD demonstrated strong generalizability on the external dataset, achieving an AUC of 0.71 for significant CAD detection and 0.75 for severe CAD detection. These results highlight the model’s robust performance across different imaging equipment and clinical settings. This study presents a novel integration of myocardial work metrics with GLS and LVEF within a multi-task learning framework for CAD diagnosis and severity grading. This approach builds on previous work and represents a significant advancement in leveraging echocardiographic data for comprehensive and automated CAD assessment (Salte et al., 2021; Upton et al., 2022).

Among all imaging techniques for diagnosing CAD, echocardiography is a common, first-line test thanks to its convenience, cost-effectiveness, and diagnostic performance. Echocardiogram-derived strain and myocardial work metrics can provide better evaluation of cardiac performance than conventional echocardiography and other diagnostic methods, and as a result, they have emerged as innovative instruments for CAD detection and evaluation (Smiseth et al., 2021; Guo et al., 2022; Guo et al., 2023). Although the measurement of strain and myocardial work from echocardiographic videos has been made a partially automatic process (Smiseth et al., 2021; Guo et al., 2022; Guo et al., 2023), it remains time-consuming and requires considerable professional expertise and hence, it is not suitable for early CAD screening in clinical practice. The recent years have seen a number of studies exploring fully automated GLS measurement using AI methods (Salte et al., 2021; Deng et al., 2022; Salte et al., 2023). Nonetheless, there have been few reports on AI models for fully automated assessment of myocardial work. The strength of the IE-CAD model lies in its ability to integrate fully automated assessment of GLS and myocardial work metrics to achieve rapid and accurate CAD diagnosis in a clinical setting.

Stress echo, a widely used non-invasive diagnostic tool, has reported AUCs ranging from 0.84 to 0.92 for detecting significant CAD, depending on interpreter experience and image quality (Picano et al., 2008; Banerjee et al., 2012). In a recent study by Upton et al. (Upton et al., 2022), an AI-assisted stress echo model achieved a specificity of 92.7% and a sensitivity of 84.4%, and an AUC of 0.93, demonstrating the potential of AI to enhance diagnostic accuracy. Resting echo interpretation, primarily based on strain parameters, has reported AUCs ranging from 0.68 to 0.80 for CAD detection (Billehaug Norum et al., 2015). Stress echo is generally more effective than resting echo in inducing myocardial ischemia and segmental wall motion abnormalities, which can lead to higher diagnostic performance. Additionally, the sample size of our study is relatively small compared to previous studies on resting and stress echo, primarily due to the labor-intensive frame-by-frame annotation required for labeling. Moving forward, we plan to expand our dataset, streamline the annotation process, and further optimize the model to enhance its diagnostic performance.

Compared to the degree of coronary artery stenosis alone, the Gensini score takes into account all major coronary arteries and their branches, providing a more comprehensive evaluation of the entire coronary tree. GLS is an indicator of the function and mechanical properties of the entire myocardium, and it can reveal areas of myocardial ischemia or injury throughout the heart. Thus, the Gensini score is closely linked to GLS. In this study, the maximal Gensini scores predicted by the IE-CAD model across the three standard apical views positively correlated with the Gensini scores determined by coronary angiography, showing a Pearson correlation coefficient of 0.545. This moderate correlation implies some prediction uncertainty. The Gensini score, while widely used in research, is not commonly adopted in clinical practice due to the lack of standardized cutoff values for defining severe CAD. However, the continuous nature of the Gensini score provides a more nuanced and precise representation of disease severity, making it particularly valuable for research and machine learning applications. In this study, we utilized the Gensini score as a continuous “soft label,” allowing the model to learn a gradient of disease severity rather than relying on arbitrary categorical thresholds. This approach enables the model to associate varying levels of CAD with subtle features in echocardiographic images, potentially improving prediction accuracy. Our future work will aim to bridge the gap between research and clinical utility, by developing standardized cutoff values and further refining our model for clinical integration. Intriguingly, the IE-CAD model classified a group of subjects as non-significant CAD who had a Gensini score of >= 15 points. This group of patients could have been underdiagnosed for CAD and therefore, they should undergo further diagnostic tests.

In this study, most subjects classified as significant CAD by the IE-CAD model also had impaired resting myocardial function. The majority of the myocardial work and strain metrics were found to be valuable parameters for diagnosing significant CAD, particularly when there was >= 70% coronary artery stenosis. Consistent with earlier findings (Edwards et al., 2019), LVEF, regional wall motion abnormalities, GWE, and global work index were identified as notable factors for identifying significant CAD. Furthermore, diabetes mellitus and plasma levels of BNP, HbA1c, and creatinine were found to provide additional predictive value in the detection of CAD. No significant differences were detected between the significant and non-significant CAD cases in other major clinical indices such as BMI, hyperlipidemia, systolic and diastolic BPs, fasting blood-glucose, uric acid, and LDL cholesterol. Notably, many subjects included in this study were taking medications at the time of enrollment, which may have influenced the levels of these clinical indices.

The IE-CAD model was developed by employing several innovative approaches. A 3DdeeplabV3+ backbone and multi-task learning were simultaneously incorporated into the core frame of its image processing network, enabling automatic tracking of the endocardial border in over 90% of cases. Previous AI-assisted echocardiogram segmentation models typically annotated only two frames of the images that correspond to the end-diastole and end-systole of the heart (Batool et al., 2023), which were not well-suited for the assessment of GLS. The IE-CAD model annotated a total of 20 frames to capture myocardial motions during a complete cardiac cycle, including 10 frames from the end-diastole to end-systole and another 10 frames from the end-systole to end-diastole. This enabled the IE-CAD model to capture the dynamic myocardial contours during a complete cardiac cycle to accurately predict GLS. A novel decoder-focused method for multi-task dense prediction, called Mixture-of-Low-Rank-Experts (MLoRE) (Yang et al., 2024), was employed to configure global task relationships in the IE-CAD model. In particular, the sparse features extracted by low-rank convolution were utilized to complement the primary features in multi-task learning to enhance the model’s predictive performance.

Future work could explore multimodal input strategies to overcome the limitations inherent in single-modality data. Integrating clinical data, such as laboratory results and ECG findings, with echocardiographic data could provide a more comprehensive assessment. Techniques such as weighted feature fusion could facilitate this integration. Additionally, incorporating more echocardiographic views (e.g., short-axis views) and modalities (e.g., color and spectral Doppler) could provide richer insights into cardiac structure, function, and hemodynamics. From a modeling perspective, exploring different algorithms for multimodal data integration, such as combining CNNs for spatial image analysis with RNNs for temporal dynamics could better capture the complexity of cardiac motion. Advanced fusion techniques, such as attention mechanisms, may further enhance the integration of multimodal data and improve diagnostic performance.

### 4.1 Limitations

This research had a number of limitations. Firstly, this study involved only two centers with a relatively small sample size, which may limit the generalizability of the findings. Secondly, the raw data were obtained on a single ultrasound machine and processed using a single data-processing algorithm before they were loaded to the IE-CAD model. This might have caused a certain level of data bias that limits the generalizability of the model. To enhance the model’s generalizability, future research should involve data from multiple centers and incorporate an adaptive learning approach that allows the model to evolve with the introduction of new data. Thirdly, the classification of “significant CAD” in this study was based on the clinical doctors’ interpretation of coronary angiography, without quantitative measurements of coronary stenosis to confirm the severity of the disease. Thus, the reliability of coronary angiography interpretation could have potentially affected the results.

In future research, new network architectures such as transformer or mamba may be incorporated into the model to improve its predictive performance. In addition, the model could be improved by automatically incorporating patients’ other diagnostic reports for rapid extraction of CAD-related features.

## 5 Conclusion

A fully automated AI model denoted IE-CAD was developed to automatically “read” the echocardiographic videos for CAD diagnosis and stenosis severity assessment. This model extracts informative ultrasound video features to yield multiple echocardiographic metrics such as LVEF, myocardial work and GLS and incorporates these factors for CAD diagnosis and coronary stenosis assessment. This model demonstrated good sensitivity and accuracy in a single center prospective trial, and it may be used for early CAD screening in a clinical setting.

## Data Availability

The raw data supporting the conclusions of this article will be made available by the authors, without undue reservation.
