# Supplementary figures and images for: Echocardiographic video-driven multi-task learning model for coronary artery disease diagnosis and severity grading
Source: Front Bioeng Biotechnol. 2025 Jul 25;13:1556748. doi: 10.3389/fbioe.2025.1556748 (PMC12331746; doi:10.3389/fbioe.2025.1556748)

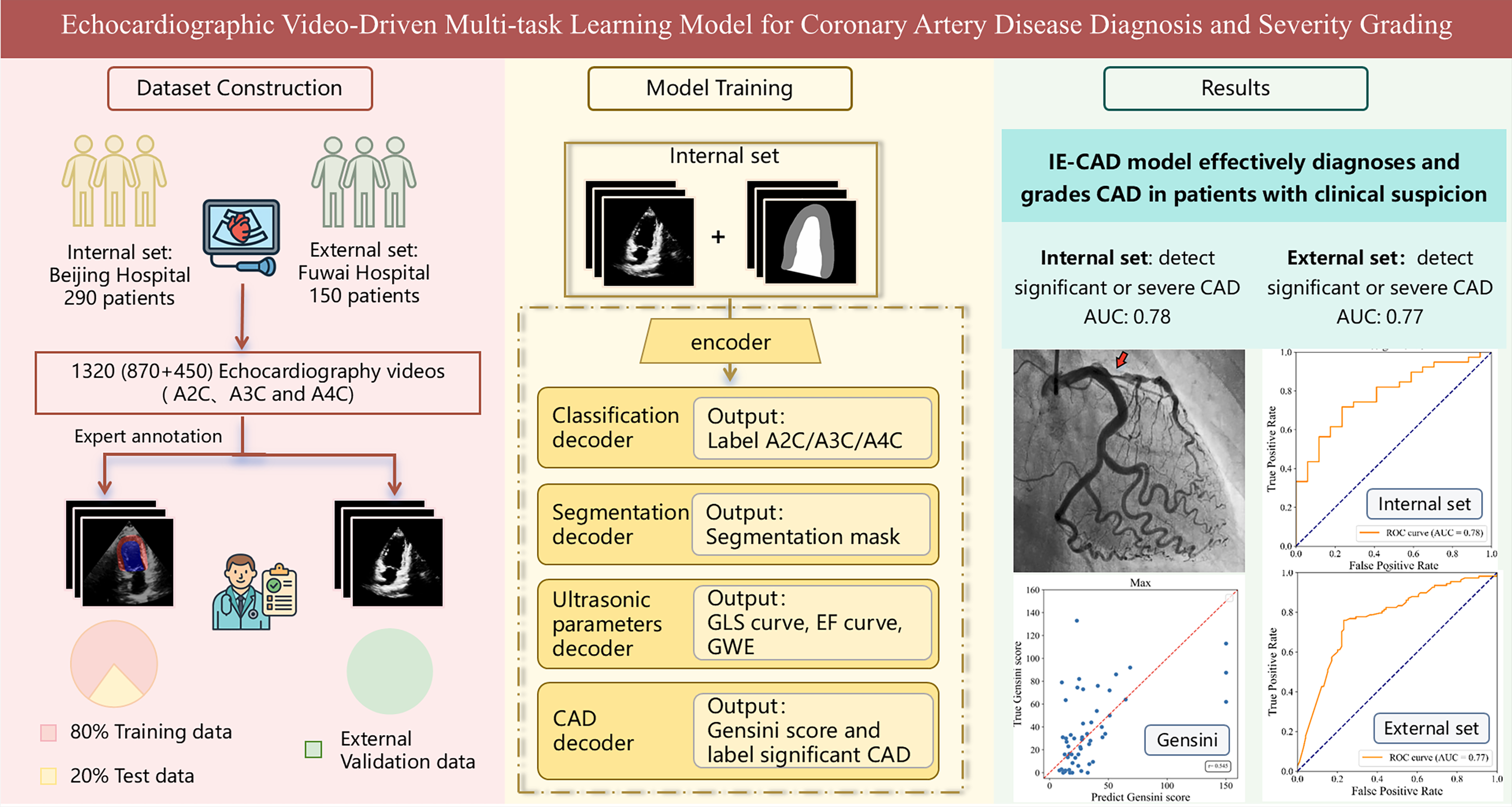

Supplement: Supplementary file 1 [file Image1.tiff]
